# Nitrapyrin addition mitigates nitrous oxide emissions and raises nitrogen use efficiency in plastic-film-mulched drip-fertigated cotton field

**DOI:** 10.1371/journal.pone.0176305

**Published:** 2017-05-08

**Authors:** Tao Liu, Yongchao Liang, Guixin Chu

**Affiliations:** 1 The Key Laboratory of Oasis Eco-agriculture, Xinjiang Production and Construction group, College of Agriculture, Shihezi University, Shihezi, P. R. China; 2 Key Laboratory of Environment Remediation and Ecological Health, Ministry of Education, College of Environmental & Resource Sciences, Zhejiang University, Hangzhou, P. R. China; Agriculture and Agri-Food Canada, CANADA

## Abstract

Nitrification inhibitors (NIs) have been used extensively to reduce nitrogen losses and increase crop nitrogen nutrition. However, information is still scant regarding the influence of NIs on nitrogen transformation, nitrous oxide (N_2_O) emission and nitrogen utilization in plastic-film-mulched calcareous soil under high frequency drip-fertigated condition. Therefore, a field trial was conducted to evaluate the effect of nitrapyrin (2-chloro-6-(trichloromethyl)-pyridine) on soil mineral nitrogen (N) transformation, N_2_O emission and nitrogen use efficiency (NUE) in a drip-fertigated cotton-growing calcareous field. Three treatments were established: control (no N fertilizer), urea (225 kg N ha^-1^) and urea+nitrapyrin (225 kg N ha^-1^+2.25 kg nitrapyrin ha^-1^). Compared with urea alone, urea plus nitrapyrin decreased the average N_2_O emission fluxes by 6.6–21.8% in June, July and August significantly in a drip-fertigation cycle. Urea application increased the seasonal cumulative N_2_O emission by 2.4 kg N ha^-1^ compared with control, and nitrapyrin addition significantly mitigated the seasonal N_2_O emission by 14.3% compared with urea only. During the main growing season, the average soil ammonium nitrogen (NH_4_^+^-N) concentration was 28.0% greater and soil nitrate nitrogen (NO_3_^-^-N) concentration was 13.8% less in the urea+nitrapyrin treatment than in the urea treatment. Soil NO_3_^-^-N and water-filled pore space (WFPS) were more closely correlated than soil NH_4_^+^-N with soil N_2_O fluxes under drip-fertigated condition (*P*<0.001). Compared with urea alone, urea plus nitrapyrin reduced the seasonal N_2_O emission factor (EF) by 32.4% while increasing nitrogen use efficiency by 10.7%. The results demonstrated that nitrapyrin addition significantly inhibited soil nitrification and maintained more NH_4_^+^-N in soil, mitigated N_2_O losses and improved nitrogen use efficiency in plastic-film-mulched calcareous soil under high frequency drip-fertigated condition.

## Introduction

As a kind of potential greenhouse gas (GHG), nitrous oxide (N_2_O) causes climate change and indirectly contributes to stratospheric ozone depletion [1], and its global warming potential (GWP) is about 298 times that of carbon dioxide (CO_2_) over a 100 yrs time horizon [2]. In China, agricultural intensification and heavy fertilizer use have led to an increase in N_2_O emission losses, the contribution of croplands to the national total of fertilizer N-induced N_2_O (FIE-N_2_O) increased from 79% to 92% only in the period of 1980–2000 [3]. Thus, reducing N_2_O emissions from agricultural sources constitutes a critical research challenge.

Agricultural N_2_O emission has long been studied, however, whether N_2_O is mainly derived from nitrification process under aerobic conditions, or from denitrification process under either anaerobic or aerobic conditions is still inconclusive. The relative contribution of each process to N_2_O is largely dependent upon soil environmental conditions (e.g. soil organic matter, pH, soil moisture, soil N status, etc.). For instance, several previous studies suggest that nitrification accounts for most N_2_O emission in well-aerated soil below 60% water-filled pore space (WFPS), whereas denitrification reaction accounts for most N_2_O emission when soil moisture exceeds 70% WFPS [4–5]. Recent studies show that denitrification prevails over nitrification during N_2_O production for irrigated dryland soils or more humid soils initially [6–7].

Many researches demonstrate that N_2_O emission can be substantially mitigated by nitrification inhibitors (NIs) through reducing the rate of ammonium nitrogen (NH_4_^+^) oxidation into nitrate nitrogen (NO_3_^-^) during nitrification, and subsequently decreasing the substrate (NO_3_^-^) concentration for denitrificationin different agro-ecosystems [8–11]. Otherwise, NIs have been used to improve crop N status [8, 12] and increase soil mineral N retention [13]. As one of the effective nitrification inhibitors, nitrapyrin (2-chloro-6-(trichloromethyl)-pyridine) exhibited some effectiveness on reducing nitrate leaching and N_2_O emission losses, improving yield and N retention in the grain and corn field or under the simulated experimental condition [13–16]. We previously showed that nitrapyrin was more effective than dicyandiamide (DCD) in controlling nitrification process, and nearly as effective as 3, 4-dimethylpyrazole phosphate (DMPP) in calcareous soil with sandy, loam and clay texture [17]. Although numerous studies have been undertaken on the influences of different NIs on N_2_O emission and nitrogen transformations, how repeated supply of nitrapyrin with urea via fertigation impacts soil N_2_O emissions, nitrogen transformation and utilization under drip-irrigated condition is still unknown. Therefore, it is necessary to clarify the influences of repeated addition of nitrapyrin on soil N_2_O emissions and its major affecting factors in plastic-film-mulched drip-fertigated cotton field.

Xinjiang, located in northwest of China, is an important agricultural area. Due to the arid climate with annual precipitation of 160.6 mm on average, conventional agriculture in this region depends heavily on irrigation. To save fresh water resource and improve water utilization efficiency, a combination of plastic film-mulching with drip irrigation has been extensively adopted in this region during the last two decades. In this system, N fertilizer is applied about 8–10 times coupling with water via the irrigation system during a growing season. As a consequence, the re-wetting of the topsoil frequently occurs, and soil N transformation process in drip irrigation condition substantially differs from that in conventional cultivation condition (i.e. flooding irrigation and single fertilizer application).

The objectives of this study were to explore the effects of nitrapyrin added to the urea solution on N_2_O emission, soil mineral nitrogen transformations and nitrogen use efficiency in the plastic-film-mulched and high frequency drip-fertigated cotton field, and ascertain the major factors impacting on N_2_O emissions in this system. The results will provide a basis for decreasing nitrogen losses and promoting efficient utilization of nitrogen fertilizer through adding nitrapyrin to urea solution in drip-fertigated cropland in arid regions.

## Materials and methods

### Study site and soil characteristic

The field trial was set up at an experiment station of Agricultural College, Shihezi University, Xinjiang, China (44°18′N, 86°02′E) in April 2013. This site has a typical temperate continental climate and the annual precipitation varies between 125.0 and 207.7 mm.

The soil at the site is a *Calcaric Fluvisals* consisting of 24.7% sand, 20.1% silt and 48.8% clay. The initial physicochemical properties are: organic matter content, 16.2 g kg^-1^; total N content, 0.92 g kg^-1^; ammonium nitrogen content, 28.0 mg kg^-1^; nitrate nitrogen content, 40.9 mg kg^-1^; soil available phosphorus, 11.0 mg kg^-1^ and available potassium, 257.5 mg kg^-1^. The soil is alkaline (pH 7.96) with bulk density of 1.42 g cm^-3^ (0–15 cm layer). The groundwater is generally used for irrigation in this region.

### Experimental design and field management

**Fig 1:**
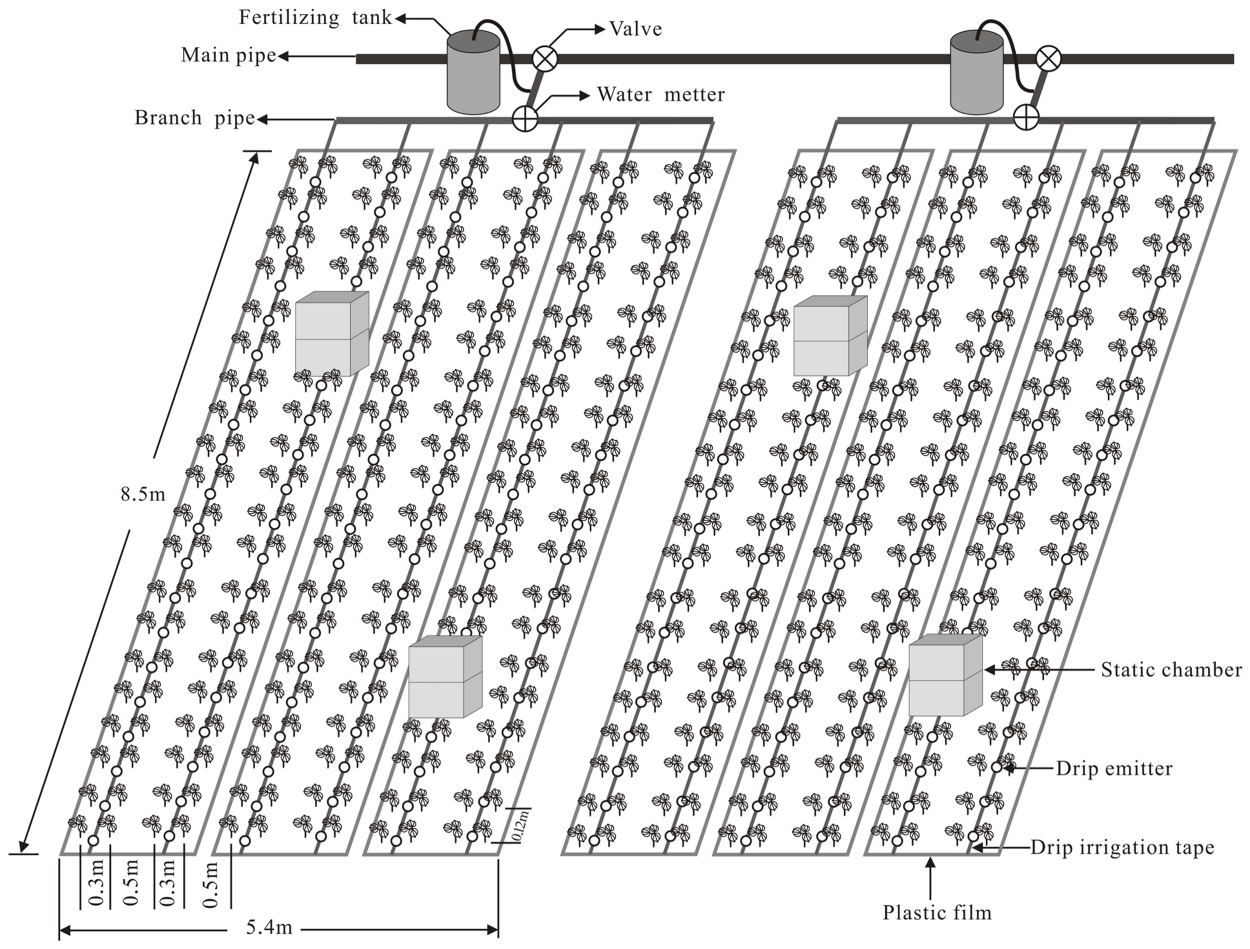
Schematic diagram of the experimental plot layout with plastic-film-mulched and drip fertigation system.

This experiment consisted of a randomized block field experiment with three treatments: (i) control (no N fertilizer), (ii) urea and (iii) urea+nitrapyrin. Each treatment was replicated three times. The area of each plot was 45.9 m^2^ (i.e. 5.4 m × 8.5 m), and 12 rows of cotton plants (*Gossypium hirsutum* cv. Xinluzao 45) were grown in each plot and the row spacing was 30 cm– 50 cm– 30 cm, and there was a 12 cm space between plants within a row (Fig 1). For each plot, a drip irrigation tape was placed in the middle of two rows, and six drip irrigation tapes were connected to a branch pipe controlled by a valve with a small fertilizing tank and a water meter installed to precisely control the irrigation volume and fertilizer rate.

A total of 465 mm irrigation water was applied to each plot in 10 split application times mainly in June, July, and August (Table 1). The nitrogen, phosphorus, and potassium fertilizers (urea and potassium dihydrogen phosphate) were dissolved in the irrigation water and applied during the first eight irrigation events. No urea was added to the irrigation water in the control treatment. The urea and urea+nitrapyrin treatments both received a total of 225 kg N ha^-1^, and all the treatments received a total of 90 kg ha^-1^ phosphorus pentoxide (P_2_O_5_) and 60 kg ha^-1^ potassium oxide (K_2_O) during the cotton growing season. Nitrapyrin, which was supplied by Zhejiang Aofutuo Chemical Ltd, P. R. China, was mixed into the irrigation water in the urea+nitrapyrin treatment at a rate of 1% urea-N. More detailed information about the schedule and rate of irrigation and fertilization was shown in Table 1.

**Table 1:** Drip fertigation schedule and the amounts of water and fertilizers.

Fertigation time (d-m)	13–06	23–06	04–07	12–07	23–07	04–08	12–08	22–08	30–08	04–09
Water (mm ha^-1^)	45	52.5	52.5	60	60	52.5	45	45	30	22.5
N (kg ha^-1^)	7.5	22.5	37.5	45	45	37.5	22.5	7.5	0	0
P_2_O_5_ (kg ha^-1^)	7.5	10.5	12	15	15	15	9	6	0	0
K_2_O (kg ha^-1^)	5	7	8	10	10	10	6	4	0	0
Nitrapyrin (kg ha^-1^)	0.075	0.225	0.375	0.45	0.45	0.375	0.225	0.075	0	0

The N rates are only for the urea and the urea+nitrapyrin treatments, nitrapyrin rates are only for the urea+nitrapyrin treatment, the rates of water, P_2_O_5_ and K_2_O are for all the treatments.

### N_2_O sampling and measurement

Gas samples for N_2_O analysis were collected using static chambers consisting of top and middle chamber part, each being 60 cm×50 cm×55 cm for L×W×H in size to adapt to the height of the growing plants, and the final effective height reaching 110 cm. There were two chambers in each plot, the base of each chamber being inserted 10 cm deep into the soil to prevent air leakage, and each chamber having two fans to mix the air. The chambers were centred over two drip irrigation emitters and covered two rows of cotton plants (Fig 1). Gas samples were collected between 9:00 and 11:00 am on the 1, 2, 3, 4, 5 and 7 d after drip fertigation in June, July and August (fertilization season); four gas samples were collected from each chamber using 50 ml disposable syringes at 0, 10, 20 and 30 min after the chambers’ coverage. Meanwhile, soil temperature was measured using soil thermometers inserted to a depth of 5 cm inside the chambers.

The N_2_O concentrations in the gas samples from the field were determined within 12 h of collection using an Agilent 7890A gas chromatograph (Agilent Technologies Ltd, USA) equipped with a 2-mm ID stainless steel column, 3-m long and packed with Porapak Q (80/100 mesh), fitted with an electron capture detector (ECD) set at 300°C. The column temperature was maintained at 40°C and the carrier gas was N_2_ (99.999% purity) at a flow rate of 30 ml min^-1^.

### Soil sampling and measurement

The determination of the initial soil organic matter, total N, available phosphorus, available potassium and pH was based on Lu [18]. In parallel with the sampling of N_2_O gas, surface soil sampling (0–15 cm in depth) was collected for the determination of soil mineral N (NH_4_^+^-N and NO_3_^-^-N) concentrations and soil moisture. The soil samples were collected at six points randomly and mixed together to form one composite sample for each plot. The soil was sieved (2 mm mesh) and 5.00 g of fresh soil was extracted with 50 ml 2 M KCl solution for 1 h by shaking on a reciprocating shaker, then the extracts were filtered through N-free quantitative filter papers and frozen at -18°C for the determination of NH_4_^+^-N and NO_3_^-^-N concentrations automatically on AA3-HR Continuous Flow Analytical System (SEAL Analytical Ltd, Germany). Soil water content was obtained by gravimetric determination after oven-drying of the samples at 105°C for 24 h to calculate WFPS.

### Nitrogen determination and yield measurement in cotton plants

Five cotton plants were randomly sampled from the harvested cotton plants and separated into stems, leaves and reproductive organs (buds, flowers, bell shells, seeds and fibres). The plant organs were oven-dried at 105°C for half an hour, and then at 75°C for another 72 hours prior to dry matter measurement. The dried samples were crushed and sieved (0.5 mm mesh) for N determination. The content of N was determined on Kjeltec 8400 Automatic Kjeldahl Nitrogen Determination Apparatus (FOSS Analytical Ltd, U.S.A.). Cotton yield was measured by weighing all of the cotton (fibres and seeds) in each plot at maturity, and the lint yield (only fibres) was calculated according to the cotton yield multiplied by lint percent.

### Estimations

N_2_O emission was calculated using Eq (1):

$$\text{F}=\frac{\text{M}}{\text{V}}\times \text{H}\times \frac{\text{P}}{{\text{P}}_{0}}\times \frac{273}{273+\text{T}}\times \frac{\text{dc}}{\text{dt}}$$ (1)

Where F (μg N m^-2^h^-1^) is N_2_O emission flux, M is the molecular weight of N_2_ in the N_2_O molecule (28 g N_2_O-N mol^−1^ for N_2_O), V is the mole volume (22.4 L mol^−1^) at 273 K and 1.013×10^5^ Pa. H (m) is the effective chamber height (1.10 m in this experiment). P is air pressure in static chamber (Pa), P_0_ is the ambient air pressure at the experimental site (1.013×10^5^ Pa), P/P_0_≈1. T is the temperature in the chamber (°C). c (ppbv) is the volume concentration of N_2_O, t (h) is the time of chamber closure, and dc/dt (ppbv h^-1^) is the rate of change in the N_2_O volume concentration in the chamber enclosure [19].

Cumulative N_2_O emission was calculated by Eq (2):

$$\text{CE}=\Sigma_{i=1}^{\text{n}}\left(\frac{{\text{F}}_{i}+{\text{F}}_{i+1}}{2}\right)\times \left({\text{t}}_{i+1}-{\text{t}}_{i}\right)\times 24$$ (2)

Where CE is N_2_O accumulative emission rate (kg N ha^-1^), F is N_2_O emission flux (μg N m^-2^h^-1^), *i* represents the *i*th times of N_2_O determination, (t_*i*+1_ − t_*i*_) is the time interval between two determinations, and n is the total determination times.

The calculation of the N_2_O emission factor (EF, %) was according to Eq (3):

$$\text{EF}\left(\%\right)=\left(\frac{{\text{CE}}_{{\text{N}}_{2}\text{O}}{}_{\left(\text{treatment}\right)}-{\text{CE}}_{{\text{N}}_{2}\text{O}}{}_{\left(\text{control}\right)}}{\text{N applied}}\right)\times 100$$ (3)

Where${\text{CE }}_{{\text{N}}_{2}\text{O }\left(\text{treatment}\right)}$is the cumulative emissions of N_2_O in the urea or urea+nitrapyrin treatment (kg N ha^-1^), ${\text{CE }}_{{\text{N}}_{2}\text{O }\left(\text{control}\right)}$is the cumulative emissions of N_2_O in the control treatment (kg N ha^-1^), and N applied is the total N amount applied to urea or urea+nitrapyrin treatment (225 kg N ha^-1^ in this experiment).

Soil water-filled pore space (WFPS) was calculated based on Eq (4):

$$\text{WFPS}\left(\%\right)=\frac{\text{SWC}\times {\text{D}}_{\text{B}}}{\left(1-\frac{{\text{D}}_{\text{B}}}{{\text{D}}_{\text{P}}}\right)}\times 100$$ (4)

Where SWC is the soil water content (%), D_B_ is the soil bulk density (g cm^-3^) and D_P_ is the soil particle density (g cm^-3^). The particle density is assumed to be 2.65 g cm^-3^, and the soil bulk density in this experiment is 1.42 g cm^-3^.

The nitrogen use efficiency (NUE) was calculated according to Eq (5):

$$\text{ NUE}\left(\%\right)=\frac{\left({\text{N uptake}}_{\left(\text{treament}\right)}-{\text{N uptake}}_{\;\left(\text{control}\right)}\right)}{\text{N input}}\times 100$$ (5)

Where N uptake_(treament)_ is the N uptake in cotton plants based on total aboveground biomass in the urea or urea+nitrapyrin treatment, N uptake_(control)_ is the N uptake in cotton plants based on total aboveground biomass in the control treatment, N input is fertilizer N application rate (225 kg N ha^-1^ in this experiment).

### Statistical analysis

The significance of differences among the different treatments in N_2_O, NH_4_^+^-N, NO_3_^-^-N, N uptake, yield, EF of N_2_O and NUE were tested via Two-way ANOVA and Duncan multiple-comparison test using SPSS 13.0 software (IBM SPSS Statistics, Inc., USA). The direct and indirect effects of the driving factors on N_2_O emissions were performed through Pearson correlation and path analysis using SPSS 13.0.

## Results and discussion

### N_2_O emission in a drip-fertigated cycle

**Fig 2:**
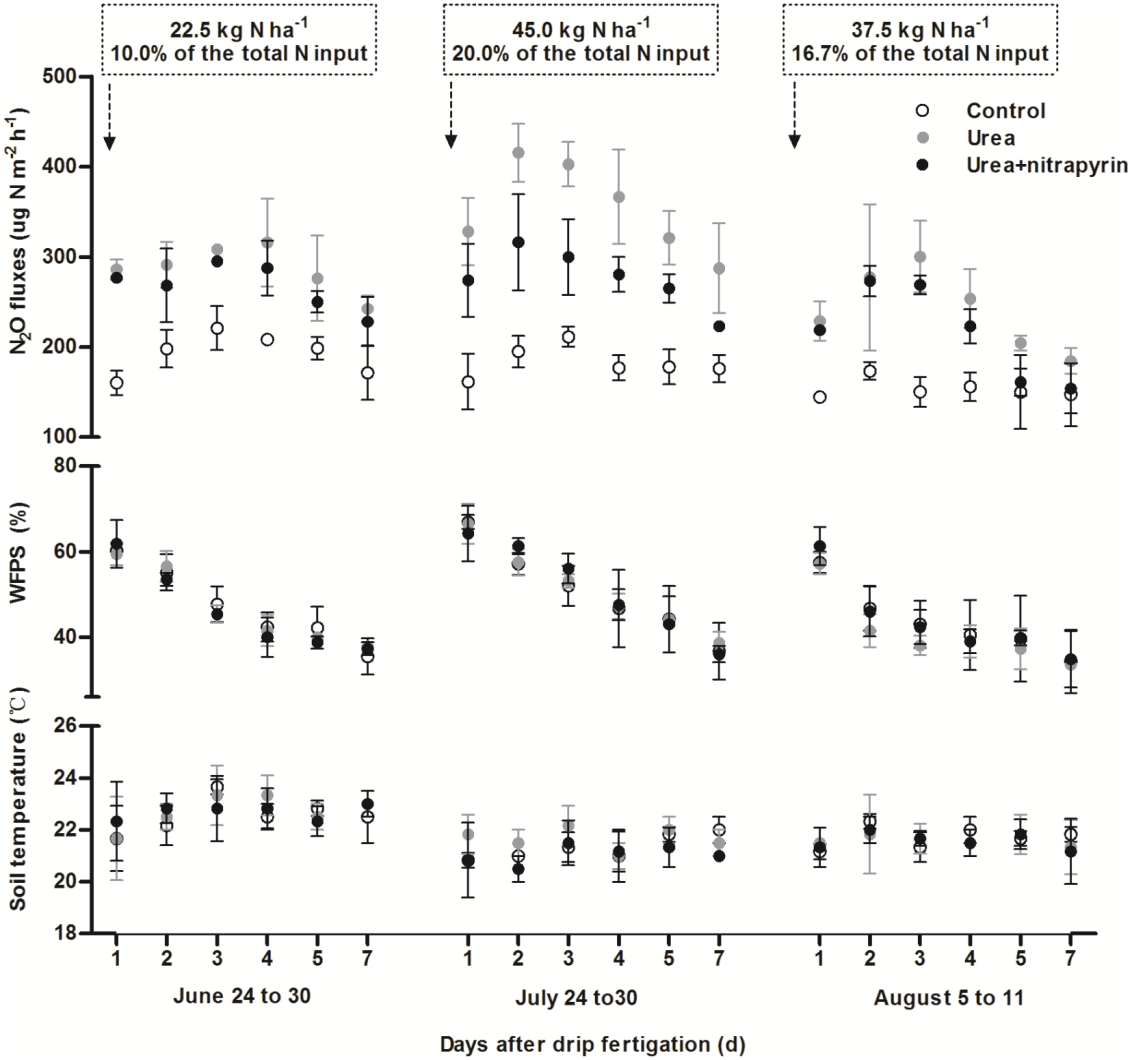
Dynamics of soil N_2_O emissions, water-filled pore space (WFPS) and soil temperature at the 5 cm depth. Error bars represent standard deviation.

Under the drip-fertigated condition, some distinctive features occurring on N_2_O emissions were attributed to frequently re-wetting and repeatedly supplying of N fertilizer, thus leading to cycling of oxi-reduction and typical featuring of N_2_O dynamics (Fig 2). It was found that soil N_2_O fluxes increased to a maximum between 2 to 4 d after fertigation and then gradually declined within each fertigation cycle across all the three months (fertilizer season) tested, which is in line with the previous reports [20–21]. The greatest emissions of N_2_O occurred in July due to the majority of water and fertilizer N addition. More N application and lower N_2_O emission were observed in August than in June, suggesting that the nearly mature cotton plants absorbed N more quickly after fertigation in August, with the result such that less N was available for conversion to N_2_O [22–23].

A marked variation in N_2_O emissions was observed in three treatments as shown in Fig 2. The average fluxes of soil N_2_O kept the lowest values of 193.0±7.9, 183.2±13.9 and 153.6±8.6 μg N m^-2^h^-1^ in June, July and August for the control treatment in seven days after drip fertigation. In the urea treatment, the average fluxes of N_2_O were increased to 286.8±4.6, 353.6±21.1 and 241.5±5.8 μg N m^-2^h^-1^, respectively, and these values were reduced to 267.8±6.1, 276.4±13.1 and 216.5±8.5 μg N m^-2^h^-1^, respectively, in urea+nitrapyrin treatment. In a fertigation cycle, nitrapyrin addition decreased N_2_O fluxes by 6.6% (*P*<0.05), 21.8% (*P*<0.01) and 10.3% (*P*<0.01), respectively, in June, July and August compared with urea only. This shows that urea plus nitrapyrin can significantly reduce soil N_2_O emission in a drip-fertigated cycle.

### Cumulative N_2_O emission during the main growing season of cotton plants

As shown in Table 2, cumulative N_2_O emissions in the urea treatment were significantly higher than in the control treatment (*P*<0.001). Compared with the urea treatment, application of urea with nitrapyrin (urea+nitrapyrin treatment) reduced cumulative N_2_O emissions by 7.1% (*P*<0.05), 21.9% (*P*<0.01) and 11.6% (*P*<0.01) in the three months tested accordingly. Urea application increased cumulative N_2_O emission by 2.4±0.1 kg N ha^-1^ during the main growing season, while nitrapyrin addition mitigated N_2_O emission by 14.3% (*P*<0.01) at the rate of 225 kg N ha^-1^.

**Table 2:** Cumulative soil N_2_O emission during the main growing season of cotton plants.

Treatments	N_2_O emissions rates (kg N ha^-1^)			Total
	June	July	August	
Control	1.13 ± 0.05 c	1.06 ± 0.07 C	0.86 ± 0.07 C	3.05 ± 0.05 C
Urea	1.76 ± 0.05 a	2.18 ± 0.14 A	1.48 ± 0.03 A	5.42 ± 0.11 A
Urea+nitrapyrin	1.64 ± 0.04 b	1.70 ± 0.07 B	1.31 ± 0.06 B	4.65 ± 0.06 B

Data were means ± SD (n = 3); Different lowercase and uppercase letters in same column mean significant differences at the level of 0.05 and 0.01, respectively according to LSD test.

At the similar rate of N application, the rate of N_2_O emissions mitigated by nitrapyrin is distinctly affected by different crop ecosystems. For instance, Xiong et al. [24] reported that the N_2_O emissions decreased by 8.8, 21.0 and 24.3% with nitrapyrin mixed with fertilizer N at the corresponding rate of 200, 300 and 400 kg N ha^-1^ in vegetable system, and a reduction rate of 50% was found by Sun et al. [25] in rice field treated with 180 and 240 kg N ha^-1^. This difference also relates to the supply of water and fertilization. Although the reduction rate of N_2_O emission by nitrapyrin in our study was lower compared with some other reports [13–16, 26], the differences in N_2_O emission between urea+nitrapyrin and urea treatment were still statistically significant (Table 2). In addition, the relatively low rate of reducing N_2_O in this study might be associated with the lower N fertilizer rate (225 kg N ha^-1^) as the typical rate of N commonly used by local cotton farmers in northwest of China was above 300 kg N ha^-1^. It is speculative that the effect of nitrapyrin in reducing N_2_O emission could be considerably higher in the condition of higher N input.

In this study, the fertilizer was applied completely during the main growing season of cotton plants (June, July and August), as a consequence, higher N_2_O emissions occurred in these months than in other growing phase. Therefore, controlling N_2_O release during the fertilizer season will contribute to the reduction of annual N_2_O emissions, especially when other conditions (e.g. initial soil physical and chemical properties, water input, environment and agronomic management) are identical; annual emission characteristic and reducing rate of N_2_O can be deduced indirectly through analyzing the seasonal cumulative N_2_O emission.

### Soil ammonium and nitrate dynamics

Soil N_2_O emission is yielded by both nitrification and denitrification processes, therefore, it may be closely related to soil NH_4_^+^-N and NO_3_^-^-N concentrations. Due to the strong nitrification usually taking place in aerobic condition, the nitrate form of mineral N is quite predominant in calcareous soils. As a result, denitrification would be prompted by the large amounts of NO_3_^-^ accumulated in soil, especially in the high soil moisture condition. For a given soil, whether nitrification or denitrification contributed more to N_2_O emission may be closely related to soil NH_4_^+^-N and NO_3_^-^-N concentrations when the initial physic-chemical factors of the tested soil are almost identical.

**Fig 3:**
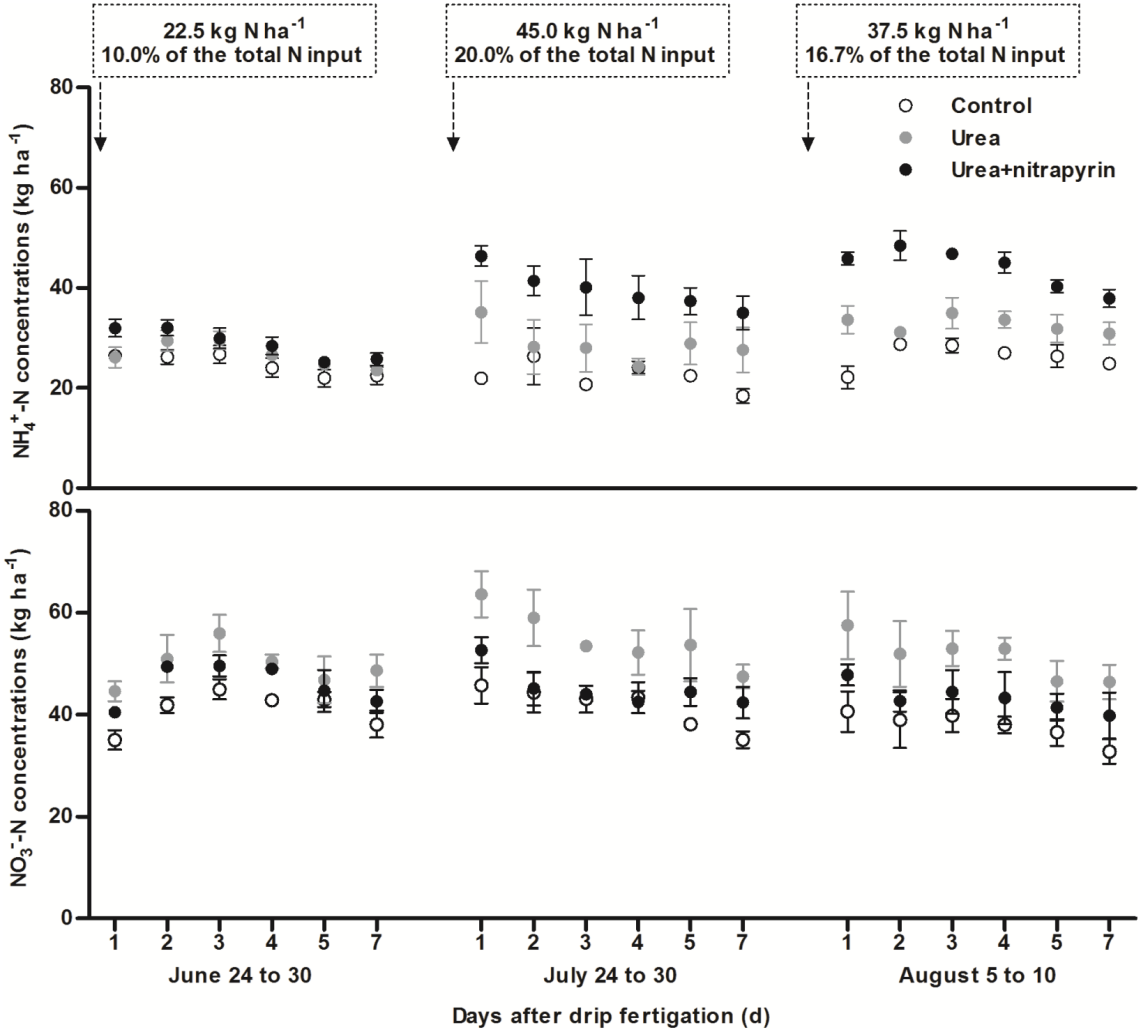
Dynamics of soil NH_4_^+^-N and NO_3_^-^-N concentrations in the 0–15 cm depth soil layer. Error bars represent standard deviation.

In this experiment, soil NH_4_^+^-N and NO_3_^-^-N concentrations were significantly greater in both urea and urea+nitripyrin treatments than in the control (Fig 3). It was found that soil NH_4_^+^-N concentrations were consistently greater in the urea+nitrapyrin treatment than in the urea treatment while the reverse was true for the NO_3_^-^-N concentrations (Fig 3). For example, the average soil NH_4_^+^-N concentrations in seven days after fertigation were 8.3% (*P*<0.05), 38.2% (*P*<0.01) and 34.8% (*P*<0.01) greater in the urea+nitrapyrin treatment than in the urea treatment in June, July and August, respectively, while soil NO_3_^-^-N concentrations were 7.3% (*P*<0.05), 17.7% (*P*<0.01) and 15.8% (*P*<0.01) less in the urea+nitrapyrin treatment than in the urea treatment in June, July and August, respectively. During the main growing season, the average soil NH_4_^+^-N concentration was 28.0% greater and soil NO_3_^-^-N concentration was 13.8% less in the urea+nitrapyrin treatment than in the urea treatment. These results indicate that nitrapyrin addition significantly inhibited soil nitrification, and shifted the soil inorganic nitrogen form from nitrate to ammonium. Earlier reports showed that soil NO_3_^-^-N concentration was 23% less and N_2_O emissions were 26 to 49% less when nitrification inhibitors (i.e., DCD and DMPP) were added to ammonium sulfate-fertilized soil [8], while soil NH_4_^+^-N concentration was increased by 41% and NO_3_^-^-N concentration was decreased by 39% in NH_4_^+^ low soils after nitrapyrin was added [26]. Our findings were less than the above reports probably due to the differences in the ecological systems, N applied rate or NI types used, etc. Overall, nitrapyrin addition significantly inhibited nitrification, and kept the soil NO_3_^-^ concentration lower, consequently decreasing the concentration of substrate used for denitrification under high-frequency-irrigation condition in this study.

### Driving factors affecting N_2_O emission

Many factors (e.g. soil temperature, WFPS, soil NH_4_^+^-N and soil NO_3_^-^-N) influence soil N_2_O emission [27–28]. In this study, the factors driving N_2_O emissions are shown in Table 3. Interestingly, significant positive Pearson correlations were noted of soil N_2_O emissions with soil NO_3_^-^-N concentrations (r = 0.628, *P*<0.001, n = 162), WFPS (r = 0.288, *P*<0.001, n = 162) and NH_4_^+^-N concentrations (r = 0.196, *P*<0.05, n = 162), but no significant correlation was found between soil N_2_O emission and soil temperature at the 5 cm depth. The effect of these factors on N_2_O emission decreased in the order of NO_3_^-^-N > WFPS >NH_4_^+^-N >soil temperature. This indicates that soil NO_3_^-^-N and WFPS are the key factors affecting N_2_O emission although NH_4_^+^-N concentrations were also significantly correlated with N_2_O fluxes as previously reported in a rape field [29].

**Table 3:** Pearson correlation and path analysis between N_2_O fluxes and soil NH_4_^+^-N concentrations, NO_3_^-^-N concentrations, water-filled pore space (WFPS), and soil temperature at the 5 cm depth (T_5cm_).

Independent variables	Pearson correlation coefficients	Direct path coefficients	Indirect path coefficients				
			NH_4_^+^-N	NO_3_^-^-N	WFPS	T_5cm_	SUM
NH_4_^+^-N	0.196*	0.139	–	0.081	0.013	-0.036	0.057
NO_3_^-^-N	0.628***	0.563	0.020	–	0.040	0.006	0.066
WFPS	0.288***	0.162	0.011	0.139	–	-0.023	0.126
Soil temperature	0.108	0.144	-0.035	0.024	-0.026	–	0.037

* and ***denote significance at the 0.05 and 0.001 probability levels-2-tailed, respectively (n = 162).

We can further determine the direct and indirect effects of the above factors on N_2_O emissions through path analysis (Table 3). In this study, soil NH_4_^+^-N concentration, soil NO_3_^-^-N concentration, WFPS, and soil temperature at the surface layer (0–5 cm in depth) directly impacted N_2_O emission, especially soil NO_3_^-^-N. Although the indirect effects of these factors on N_2_O were much weaker than the direct effects, it was still found that WFPS also regulated N_2_O emission through affecting NO_3_^-^-N. From the result of the Pearson and path analysis, N_2_O emission was largely co-regulated by both soil NO_3_^-^-N concentrations and WFPS under this film-mulched drip-fertigated condition. In addition, it was more difficult to obtain a significant correlation between N_2_O and soil temperature probably due to low soil temperature (<25°C) [30] and relatively narrow range of soil temperature (20–24°C) in this test.

N_2_O can be produced via both nitrification and denitrification when soil WFPS is at an intermediate levels (i.e. 30% -70%) [4–5, 31]. It was discovered by Abalos et al. [27] that frequent irrigation (once per week) could maintain soil WFPS in the upper soil layer at a higher level than did conventional flood irrigation, which significantly stimulated denitrification. As a result, N_2_O emission was greater with frequent drip irrigation than with conventional irrigation. In our study, frequent rewetting with drip irrigation maintained soil WFPS between 31.2% and 67.0% (Fig 2), suggesting that both nitrification and denitrification could contribute to N_2_O emission. Combining with the strong relationship between N_2_O and soil NO_3_^-^-N in Pearson correlation and path analysis, we could preliminarily speculate that more N_2_O was produced via denitrification in this plastic-film-mulched drip-fertigated system. However, more precise future work is needed to determine whether denitrification dominates nitrification during frequent rewetting of soil due to drip-fertigation in plastic-film-mulched calcareous soil.

### The effect of nitrapyrin on the EF of soil N_2_O, N uptake, yield and NUE

A wide range of N_2_O emission factors (0.01–3.7%) is shown in different countries and regions [32–35] due to the differences in soil, environment, temperature, crops and management practices. In a rice-winter wheat rotation system in southeast China, the fertilizer-induced emission factor for N_2_O averaged 1.02% during the rice season, 1.65% during the wheat season, and 1.25% during the whole annual cycle [36]. Scheer et al. [37] reported an average N_2_O emission factor of 1.48% in an irrigated cotton field in Uzbekistan (the arid deserts of Aral Sea Basin). Liu et al. [38–39] also showed that direct N_2_O emission factors in a cotton field varied between 0.9% and 2.2%. However, information is still lacking on soil N_2_O emission under plastic-film-mulched drip-fertigated condition. Similar to the previous results in China [36, 38–40], the EF of N_2_O in this study was 1.05% in the urea treatment during the main growing season of cotton plants, compared to 0.71% when nitrapyrin was added to the urea solution (Table 4); nitrapyrin addition decreased the EF of N_2_O by 32.4%. In view of the fertilizer N being applied completely in three months (the main growing seasons), it can be speculated that the annual EF of N_2_O would be higher than the seasonal EF based on the continued effectiveness of fertilizer N, and the difference between the seasonal and annual EF would not be too much on account of lower N input (225 kg N ha^-1^) in this experiment.

**Table 4:** Yield, N uptake, emission factor (EF) of N_2_O and nitrogen use efficiency (NUE).

Treatments	Lint yield (t ha^-1^)	Aboveground N uptake (kg ha^-1^)	EF of N_2_O (%)	NUE (%)
Control	1.91 ± 0.10 b	236.92 ± 5.02 b	-	-
Urea	2.48 ± 0.19 a	356.95 ± 12.39 a	1.05 ± 0.04 B	53.35 ± 3.28 b
Urea+nitrapyrin	2.58 ± 0.18 a	369.79 ± 8.68 a	0.71 ± 0.03 A	59.05 ± 1.63 a

Data were means ± SD (n = 3); Different lowercase and uppercase letters in same column mean significant differences at the level of 0.05 and 0.01, respectively according to LSD test.

Compared with urea alone, the yield and nitrogen uptake in the urea+nitrapyrin treatment were increased by 4.1 and 3.6% (*P*>0.05), respectively. The yield in this study was lower than reported earlier [13, 41–42]. Our findings were more similar to the report by Crawford and Chalk [43] who found that there was no significant difference in yield between fertilizer N plus nitrapyrin and N applied only. Under the tested condition of drip-irrigated plot with input of 225 kg N ha^-1^, the relatively high NUE (>50%) was gained, and more importantly, nitrapyrin addition increased NUE by 10.7% (*P*<0.05). Zhang et al. [44] observed that nitrapyrin raised NUE by 12.6% (*P*<0.05) in an intensively managed vegetable cropping system, and a similar finding was reported by Sun et al. [25] in a rice field. Clearly, these discrepancies were probably attributed to the differences indifferent crops tested as cotton crop tested in this study is less responsive to the enhanced ammonium nutrition caused by nitrificantion inhibitors, than wheat, corn or rice used in previous studies [13, 41–42].

## Conclusions

In plastic-film-mulched and high frequency drip-fertigated system, repeated use of the nitrapyrin significantly inhibited nitrification, shifted the ratio of soil NH_4_^+^ to NO_3_^-^, decreased N_2_O emission by 14.3% and EF of N_2_O by 32.4%, increased NUE by 10.7% in the calcareous cotton field amended with urea at the rate of 225 kg N ha^-1^. NO_3_^-^ was the key factor impacting on N_2_O emissions in this system; nitrapyrin addition decreased the concentration of denitrifying substrate (NO_3_^-^) through inhibiting nitrification process and also reduced N_2_O emission from denitrification process. Taken together, the repeated application of nitrapyrin through drip fertigation is an efficient approach to reducing N losses and promoting nitrogen use efficiency in plastic-film-mulched drip-fertigated calcareous soil in arid areas.

## Supporting information

S1 Data Data for the figures and tables. (XLSX)

## References

[1] Ravishankara AR, Daniel JS, Portman RW. Nitrous oxide (N_2_O): the dominant ozone-depleting substance emitted in the 21st century. Science 2009; 326: 123–125. 10.1126/science.1176985 19713491

[2] IPCC, Climate change 2007: the physical science basis In: Solomon S, Qin D, Manning M, Chen Z, Marquis M, Averyt K, et al (Eds.). Contribution of Working Group I to the Fourth Assessment Report of the Intergovernmental Panel on Climate Change. Cambridge University Press, Cambridge, United Kingdom; New York, NY, USA, 2007; 996.

[3] Zou JW, Lu YY, Huang Y. Estimates of synthetic fertilizer N-induced direct nitrous oxide emission from Chinese croplands during 1980–2000. Environ. Pollut. 2010; 158 (2): 631–635. 10.1016/j.envpol.2009.08.026 19762135

[4] Pihlatie M, Syväsalo E, Simojoki A, Esala M, Regina K. Contribution of nitrification and denitrification to N_2_O production in peat, clay and loamy sand soils under different soil moisture conditions. Nutr. Cycl. Agroecosyst. 2004; 70:135–141.

[5] Bateman EJ, Baggs EM. Contributions of nitrification and denitrification to N_2_O emissions from soils at different water-filled pore space. Bio. Fertil. Soil 2005; 41: 379–388.

[6] Scheer C, Wassmann R, Butterbach-Bahl K, Lamers JPA, Martius C. The relationship between N_2_O, NO, and N_2_ fluxes from fertilized and irrigated dryland soils of the Aral Sea Basin, Uzbekistan. Plant Soil 2009; 314: 273–283.

[7] Vilain G, Garnier J, Decuq C, Lugnot M. Nitrous oxide production from soil experiments: denitrification prevails over nitrification. Nutr. Cycl. Agroecosyst. 2014; 98(2):1–18.

[8] Weiske A, Benckiser G, Herbert T, Ottow JCG. Influence of the nitrification inhibitor 3, 4-dimethylpyrazole phosphate (DMPP) in comparison to dicyandiamide (DCD) on nitrous oxide emissions, carbon dioxide fluxes and methane oxidation during 3 years of repeated application in field experiments. Biol. Fertil. Soils. 2001; 34: 109–117.

[9] Merino P, Mene´ndez S, Pinto M, Gonza´lez-Murua C, Estavillo JM. 3, 4-Dimethylpyrazole phosphate reduces nitrous oxide emissions from grassland after slurry application. Soil Use Manage. 2005; 21: 53–57.

[10] Zaman M, Saggar S, Blennerhassett JD, Singh J. Effect of urease and nitrification inhibitors on N transformation gaseous emissions of ammonia and nitrous oxide, pasture yield and N uptake in grazed pasture system. Soil Biol. Biochem. 2009; 41: 1270–1280.

[11] Di HJ, Cameron KC. How does the application of different nitrification inhibitors affect nitrous oxide emissions and nitrate leaching from cow urine in grazed pastures? Soil Use Manage. 2012; 28: 54–61.

[12] Freney JR, Chen DL, Mosier AR, Rochester IJ, Constable GA, Chalk PM. Use of nitrification inhibitors to increase fertilizer nitrogen recovery and lint yield in irrigated cotton. Fertil. Res.1993; 34: 37–44.

[13] Wolt JD. A meta-evaluation of nitrapyrin agronomic and environmental effectiveness with emphasis on corn production in the Midwestern USA. Nutr. Cycl. Agroecosyst. 2004; 69: 23–41.

[14] Aulakh MS, Rennie DA, Paul EA. Acetylene and N-Serve effects upon N_2_O emissions from NH_4_ and NO_3_ treated soils under aerobic and anaerobic conditions. Soil Bio. Biochem. 1984; 16: 351–356.

[15] Bronson KF, Mosier AR, Bishnoi SR. Nitrous oxide emissions in irrigated corn as affected by nitrification inhibitors. Soil Sci. Soc. Am. J. 1992; 56: 161–165.

[16] Parkin TB, Hatfield JL. Influence of nitrapyrin on N_2_O losses from soil receiving fall-applied anhydrous ammonia. Agric. Ecosyst. Environ. 2010; 136: 81–86.

[17] Liu T, Liang YC, Chu GX, Ma D, Liu Q, Wang J. Effect comparison of three different types of nitrification inhibitors (DCD, DMPP and nitrapyrin) in calcareous soils. Soils 2011; 43(5): 758–762.

[18] Lu RK. Analytical methods of soil agrichemistry. Beijing: China Agricultural Science and Technology Press, 2000.

[19] Zheng XH, Wang MX, Wang YS, Shen RX, Gou J, Li J, et al Impacts of soil moisture on nitrous oxide emission from croplands: a case study on the rice-based agro-ecosystem in Southeast China. Chemosphere Global Change Sci. 2000; 2: 204–227.

[20] Majumdar D, Pathak H, Kumar S, Jain MC. Nitrous oxide emission from a sandy loam Inceptisol under irrigated wheat in India as influenced by different nitrification inhibitors. Agric. Ecosyst. Environ. 2002; 91: 283–293.

[21] Bhatia A, Sasmal S, Jain N, Pathak H, Kumar R, Singh A. Mitigating nitrous oxide emission from soil under conventional and no-tillage in wheat using nitrification inhibitors. Agric. Ecosyst. Environ. 2010; 136: 247–253.

[22] Duxbury JM, Bouldin DR, Tate RL. Emission of nitrous oxide from soils. Nature 1982; 298: 462–464.

[23] Maljanen M, Liikanen A, Silvola J, Martikainen PJ. Nitrous oxide emission from boreal organic soil under different land-use. Soil Biol. Biochem. 2003; 35: 689–700.

[24] Xiong W, Xia YQ, Zhou W, Yan XY. Relationship between nitrogen application rate and nitrous oxide emission and effect of nitrification inhibitor in vegetable farming system. Acta Pedologica Sinica. 2013; 50: 743–751.

[25] Sun HJ, Zhang HL, Powlson D, Min J, Shi WM. Rice production, nitrous oxide emission and ammonia volatilization as impacted by the nitrification inhibitor2-chloro-6-(trichloromethyl)-pyridine. Field Crop Res. 2015; 173: 1–7.

[26] López NI, Austin AT, Sala OE, Méndez BS. Controls on nitrification in a water-limited ecosystem: Experimental inhibition of ammonia-oxidizing bacteria in the Patagonian steppe. Soil Biol. Biochem. 2003; 35(12): 1609–1613.

[27] Davidson EA, Swank WT. Environmental parameters regulating gaseous nitrogen losses from two forested ecosystems via nitrification and denitrification. Appl. Environ. Microbiol. 1986; 52: 1287–1292. 16347234 10.1128/aem.52.6.1287-1292.1986 PMC239223

[28] Abalos D, Sanchez-Martin L, Garcia-Torres L, Groenigen JW, Vallejo A. Management of irrigation frequency and nitrogen fertilization to mitigate GHG and NO emissions from drip-fertigated crops. Sci. Total. Environ. 2014; 490: 880–888. 10.1016/j.scitotenv.2014.05.065 24908647

[29] Lin S, Iqbal J, Hu R, Wu J, Zhao J, Ruan L, et al Nitrous oxide emissions from rape field as affected by nitrogen fertilizer management: A case study in Central China. Atmos. Environ. 2011; 45: 1775–1779.

[30] Maljanen M, Martikkala M, Koponen HT, Virkajärvi P, Martikainen PJ. Fluxes of nitrous oxide and nitric oxide from experimental excreta patches in boreal agricultural soil. Soil Biol. Biochem. 2007; 39: 914–920.

[31] Skiba U, Smith KA, Fowler D. Nitrification and denitrification as sources of nitric oxide and nitrous oxide in a sandy loam soil. Soil Biol. Biochem. 1993; 25: 1527–1536.

[32] Brown L, Brow SA, Jarvis SC, Syed B, Goulding KWT, Phillips VR. An inventory of nitrous oxide emissions from agriculture in the UK using the IPCC methodology: emission estimate, uncertainty and sensitivity analysis. Atmos. Environ. 2001; 35: 1439–1449.

[33] Velthof GL, Kuikman PJ, Oenema O. Nitrous oxide emission from soils amended with crop residues. Nutr. Cycl. Agroecosyst. 2002; 62: 249–261.

[34] Ding WX, Cai Y, Cai ZC, Yagi K, Zheng XH. Nitrous oxide emissions from an intensively cultivated maize–wheat rotation soil in the North China Plain. Sci. Total Environ. 2007; 373: 501–511. 10.1016/j.scitotenv.2006.12.026 17229455

[35] Merino P, Artetxe A, Castellón A, Menéndez S, Aizpurua A, Estavillo JM. Warming potential of N_2_O emissions from rapeseed crop in Northern Spain. Soil Till. Res. 2012; 123: 29–34.

[36] Zou JW, Huang Y, Lu YY, Zheng XH, Wang YS. Atmospheric Direct emission factor for N_2_O from rice–winter wheat rotation systems in southeast China. Atmos. Environ. 2005; 39: 4755–4765.

[37] Scheer C, Wassmann R, Kienzler K, Ibragimov N, Eschanov R. Nitrous oxide emissions from fertilized, irrigated cotton (*Gossypium hirsutum L*.) in the Aral Sea Basin, Uzbekistan: Influence of nitrogen applications and irrigation practices. Soil Biol. Biochem. 2008; 40: 290–301.

[38] Liu CY, Zheng XH, Zhou ZX, Han SH, Wang YH, Wang K, et al Nitrous oxide and nitric oxide emissions from an irrigated cotton field in Northern China. Plant Soil.2010; 332:123–134.

[39] Liu CY, Yao ZH, Wang K, Zheng XH. Three-year measurements of nitrous oxide emissions from cotton and wheat-maize rotational cropping systems. Atmos. Environ. 2014; 6: 201–208.

[40] Zhang Q, Ju XT, Zhang FS. Re-estimation of direct nitrous oxide emission from agricultural soils of China via revised IPCC 2006 guideline method. Chin J Eco-Agric. 2010; 18(1): 7–13.

[41] Swezey ANL, Turner GO. Crop experiments on the effect of 2-chloro-6-(trichloromethyl)-pyridine for the control of nitrification of ammonium and urea fertilizer. J Agron Crop Sci. 1962; 54: 532–535.

[42] Huber DM, Warren HL, Nelson DW. Nitrification inhibitors: new tools for food production. Bioscience.1977; 27(8): 523–529.

[43] Crawford DM, Chalk PM. Sources of N uptake by wheat (*Triticum aestivum L*.) and N transformations in soil treated with a nitrification inhibitor (nitrapyrin). Plant Soil 1993; 149(1): 59–72.

[44] Zhang M, Fan CH, Li QL, Li B, Zhu YY, Xiong ZQ. A 2-yr field assessment of the effects of chemical and biological nitrification inhibitors on nitrous oxide emissions and nitrogen use efficiency in an intensively managed vegetable cropping system. Agric. Ecosyst. Environ. 2015 201: 43–50.

